# Conflict Detection in a Sequential Decision Task Is Associated with Increased Cortico-Subthalamic Coherence and Prolonged Subthalamic Oscillatory Response in the β Band

**DOI:** 10.1523/JNEUROSCI.0572-21.2022

**Published:** 2022-06-08

**Authors:** E. Zita Patai, Thomas Foltynie, Patricia Limousin, Harith Akram, Ludvic Zrinzo, Rafal Bogacz, Vladimir Litvak

**Affiliations:** ^1^Medical Research Council Brain Network Dynamics Unit, Oxford University, Oxford OX1 3TH, United Kingdom; ^2^Wellcome Centre for Human Neuroimaging, University College London Queen Square Institute of Neurology, London WC1N 3AR, United Kingdom; ^3^Functional Neurosurgery Unit, The National Hospital for Neurology and Neurosurgery and Department of Clinical and Movement Neurosciences, University College London Queen Square Institute of Neurology, London WC1N 3BG, United Kingdom; ^4^Department of Psychology, School of Biological and Behavioral Sciences, Queen Mary University, London E1 4NS, United Kingdom

**Keywords:** coherence, conflict, DBS, dorsal premotor cortex, evidence integration, subthalamic nucleus, human

## Abstract

Making accurate decisions often involves the integration of current and past evidence. Here, we examine the neural correlates of conflict and evidence integration during sequential decision-making. Female and male human patients implanted with deep-brain stimulation (DBS) electrodes and age-matched and gender-matched healthy controls performed an expanded judgment task, in which they were free to choose how many cues to sample. Behaviorally, we found that while patients sampled numerically more cues, they were less able to integrate evidence and showed suboptimal performance. Using recordings of magnetoencephalography (MEG) and local field potentials (LFPs; in patients) in the subthalamic nucleus (STN), we found that β oscillations signaled conflict between cues within a sequence. Following cues that differed from previous cues, β power in the STN and cortex first decreased and then increased. Importantly, the conflict signal in the STN outlasted the cortical one, carrying over to the next cue in the sequence. Furthermore, after a conflict, there was an increase in coherence between the dorsal premotor cortex and STN in the β band. These results extend our understanding of cortico-subcortical dynamics of conflict processing, and do so in a context where evidence must be accumulated in discrete steps, much like in real life. Thus, the present work leads to a more nuanced picture of conflict monitoring systems in the brain and potential changes because of disease.

**SIGNIFICANCE STATEMENT** Decision-making often involves the integration of multiple pieces of information over time to make accurate predictions. We simultaneously recorded whole-head magnetoencephalography (MEG) and local field potentials (LFPs) from the human subthalamic nucleus (STN) in a novel task which required integrating sequentially presented pieces of evidence. Our key finding is prolonged β oscillations in the STN, with a concurrent increase in communication with frontal cortex, when presented with conflicting information. These neural effects reflect the behavioral profile of reduced tendency to respond after conflict, as well as relate to suboptimal cue integration in patients, which may be directly linked to clinically reported side-effects of deep-brain stimulation (DBS) such as impaired decision-making and impulsivity.

## Introduction

Whether it is deciding which method of transportation to take to get to work most efficiently or which horse to bet on to maximize monetary gain, humans are constantly integrating noisy evidence from their environment and past experience, to optimize their decisions. Often the information comes at intervals, thus necessitating a system that can track incoming signals over time and only commit to making a choice after sufficient evidence has been integrated (Ratcliff, 1978; Busemeyer and Townsend, 1993; Usher and McClelland, 2001), a process that has been proposed to rely on the cortico-basal-ganglia circuit (Bogacz et al., 2010). Research in human patients with implanted electrodes for clinical deep-brain stimulation (DBS) treatment has pointed to the role of the subthalamic nucleus (STN) of the basal ganglia as a decision gate-keeper. The STN is postulated to set the decision threshold in the face of conflicting information by postponing action initiation until the conflict is resolved (Frank, 2006). As predicted by the model, STN activity is increased for high conflict trials and STN-DBS affects decision-making in the face of conflicting evidence (Frank et al., 2007; Coulthard et al., 2012; Green et al., 2013). Furthermore, the decision threshold correlated specifically with changes in STN θ oscillatory power (Cavanagh et al., 2011; Herz et al., 2016). Recent evidence has also pointed to the role of β oscillations during conflict (Zavala et al., 2018). Thus, oscillatory activity, primarily in the θ and β bands, in the basal ganglia, reflects immediate inhibition to motor output during situations involving conflict (Frank, 2006), whether it is the response, sensory, or cognitive uncertainty (Bonnevie and Zaghloul, 2019).

The majority of previous studies in the STN employed paradigms in which the putative processes of conflict detection and setting of decision threshold happened in close temporal proximity. For example, in previously used paradigms such as the flanker task (Zavala et al., 2015), go-no-go (Alegre et al., 2013; Benis et al., 2014), and Stroop task (Brittain et al., 2012) evidence was presented simultaneously. Although STN activity was also studied in random dot motion paradigm that required evidence accumulation over time (Herz et al., 2018), it was unknown exactly what sensory evidence was presented when, on individual trials, because of the noisy nature of stimuli. As a result, previous studies do not allow us to fully disentangle the neural correlates of ongoing evidence accumulation and conflict during decision-making. In particular, it is not clear what kind of conflicting information during evidence accumulation the STN responds to: does it respond to a local conflict, when a new piece of information does not match single previous piece in the sequence, or global conflict, when a new piece of information does not match overall evidence from the entire trial?

An important role in shaping the STN activity is played by the interaction between the cortical circuits and the STN. However, the nature and cortical locus of this interaction has only been examined in a handful of studies. Resting-state coherence between the STN and ipsilateral frontal cortex has shown a peak in the β band in human patients (Litvak et al., 2011a; West et al., 2020) as well as rodent models of Parkinson's disease (Magill et al., 2004; West et al., 2018). Additionally, coherence in the θ band from frontal sites (as measured with electroencephalography) to the STN increased during a conflict detection task (Zavala et al., 2014, 2016).

**Figure 1.**
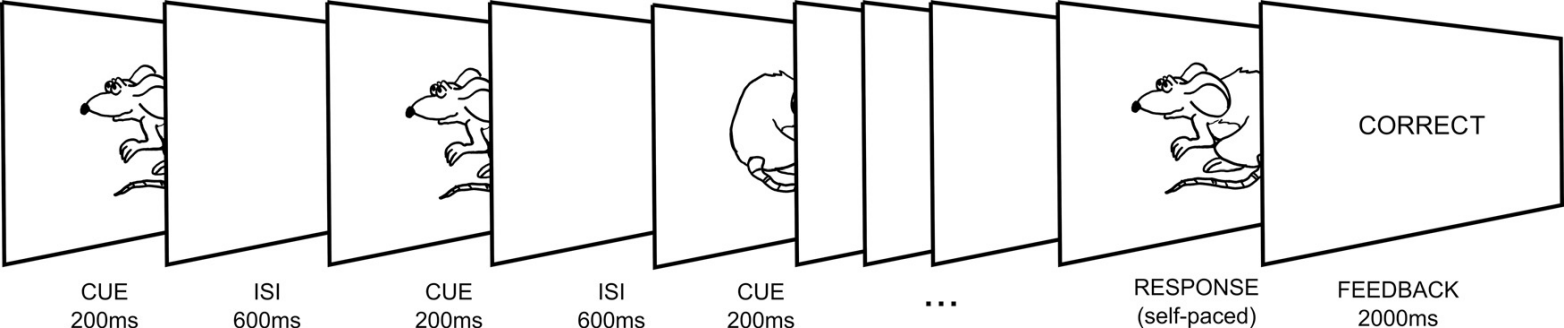
Expanded judgement task. Participants performed a version of an evidence integration task, with two key elements: (1) the cues were presented sequentially within the trial rather than simultaneously, which allowed us to examine evidence accumulation over time; and (2) the trial duration, i.e., number of cues sampled, was up to the participants, who responded when they felt they had received enough information to make a decision. Participants were required to guess the likely direction (left or right) the mouse “would run” in. Each cue was 70% valid, i.e., they represented the correct direction 70% of the time if they were to be treated in isolation.

To precisely characterize how the neural activity in cortex and the STN changes during the process of evidence accumulation, we recorded STN local field potential (STN-LFP) simultaneously with whole-head magnetoencephalography (MEG) while Parkinson's disease patients performed an expanded judgment task (Leimbach et al., 2018). Here, cues are presented at discrete intervals, and evidence for the correct answer develops as the participant samples and integrates multiple cues over the course of the trial (Fig. 1). This paradigm allowed us to investigate how behavioral and neural responses depend on the continual unfolding of evidence extended in time, determine what kind of conflicting information the STN responds to, and test predictions of computational models.

## Materials and Methods

### Participants

We tested 15 patients with a clinical diagnosis of Parkinson's disease (14 male, mean age: 59, range 47–71, two left-handers), following electrode implantation for DBS treatment, before full closure of the scalp, thus allowing for intracranial recordings of the STN (all bilateral recordings, except 1 patient right unilateral and 1 patient with three contacts in the left STN and only two on the right, this patient was also subsequently diagnosed with multiple systems atrophy). Among tested patients, 11 had medtronic 3389 electrodes, while four had Boston Vercise directional leads. The surgical procedures are described in detail in (Foltynie et al., 2011). All patients were assessed on medication (mean levodopa equivalent dosage 1272 mg, range: 500–1727.5 mg). Unified Parkinson's Disease Rating Scale (UPDRS) part 3 scores were 39.6 ± 14 (mean ± SD, range: 18–61) when OFF medication, and 15.4 ± 6.5 (range: 7–30) when ON medication. None of the patients had cognitive impairment [Mini–Mental State Examination (MMSE) scores: mean 28.8, range: 26–30, one patient score missing], clinical depression, or apathy. Two patients were excluded from the analysis because of poor performance of the task (see below, Task). We recruited 13 age and gender matched controls (12 male, mean age: 57, range 44–70, two left-handers). The patient study was approved by the United Kingdom National Research Ethics Service Committee for South Central Oxford and the control study was covered by University College London Ethics Committee approval for minimum risk MEG studies of healthy human cognition. All participants gave written informed consent. Patients did not receive financial compensation and the controls were compensated for their time according to our center's standard hourly rate.

### Surgical procedure

Bilateral DBS implantation was performed under general anesthesia using a stereotactic (Leksell frame G, Elekta Solutions AB, Stockholm, Sweden) MRI-guided and MRI-verified approach without microelectrode recording as detailed in previous publications (Holl et al., 2010; Foltynie et al., 2011). Two stereotactic, preimplantation scans were acquired, as part of the surgical procedure, to guide lead implantation; a T2-weighted axial scan (partial brain coverage around the STN) with voxel size of 1.0 × 1.0 mm^2^ (slice thickness = 2 mm) and a T1-weighted 3D-MPRAGE scan with a (1.5 mm)^3^ voxel size on a 1.5T Siemens Espree interventional MRI scanner. Three-dimensional distortion correction was conducted using the scanner's built-in module. Target for the deepest contact was selected at the level of maximal rubral diameter (∼5 mm below the AC-PC line). To maximize DBS trace within the STN, the target was often chosen 1.5–2 mm posterolateral to that described by Bejjani (Bejjani et al., 2000). Stereotactic imaging was repeated following lead implantation to confirm placement.

### Task

To investigate the neural basis of evidence accumulation over time, we used the expanded judgment task (Fig. 1; similar to the task previously used by Leimbach et al., 2018). Participants were shown a series of images of a mouse facing either left or right. Cues were presented for 200 ms, with an interstimulus interval (ISI) of 600 ms, so there was 800 ms interval from one onset to another, to which we refer as stimulus onset asynchrony (SOA). Participants were required to judge in which direction the mouse “would run”, based on the probabilities extracted from a series of sequential cue images, and then respond accordingly. The validity of the cues was 70%, such that each cue (left or right mouse) represented the correct choice 70% of the time. The two directions were equally likely across trials, thus the chance level in the task was 50%. If the participants responded based on one of the cues only, without accumulating information over time, then their expected success rate would be 70%. Responses were made by pressing a button with the thumb of the congruent hand after a self-chosen number of cues, when the participant felt they had enough evidence to make a decision. Before the recording, the participants underwent a short training session where they were first asked to respond only after seeing a set number of stimuli (between two and ten) and then told that for the main experiment they will decide themselves how many stimuli to observe. This was to ensure that participants chose to respond based on accumulating evidence from a sequence of images rather than just the first stimulus. Participants performed up to 200 trials (patients: 168 ± 11; controls: 200 each, except one control who completed 150 trials). Two patients were excluded from the analysis because of poor performance of the task (accuracy at chance level).

### Recording and analysis

Participants performed the task while seated in a whole-head MEG system (CTF-VSM 275-channel scanner, Coquitlam, Canada). For patients, STN-LFP, electrooculography (EOG), and electromyography (EMG) recordings were also obtained using a battery-powered and optically isolated EEG amplifier (BrainAmp MR, Brain Products GmbH, Gilching, Germany). STN-LFP signals were recorded referenced to a common cephalic reference (right mastoid).

All preprocessing was performed in SPM12 (v. 7771, http://www.fil.ion.ucl.ac.uk/spm/; Litvak et al., 2011b), and spectral analysis and statistical tests were performed in Fieldtrip (http://www.ru.nl/neuroimaging/fieldtrip/; Oostenveld et al., 2011) using the version included in SPM12.

STN-LFP recordings were converted offline to a bipolar montage between adjacent contacts (three bipolar channels per hemisphere; 01, 12, and 23) to limit the effects of volume conduction from distant sources (for more details, see Litvak et al., 2010; Oswal et al., 2016b). Four of the patients had segmented DBS leads (Vercise DBS directional lead, Boston Scientific). In these cases, we averaged offline the signals from the three segments of each ring and treated them as a single ring contact. Thus, for each participant, we had a total of three STN EEG channels in each hemisphere (except for two participants: one with right side electrodes only, thus three channels, and one with one contact on the right excluded because of extensive noise, thus five channels). The LFP data were downsampled to 300 Hz and high-pass filtered at 1 Hz (Butterworth fifth order, zero phase filter).

A possibly problematic but unavoidable feature of our task was that the stimuli were presented at relatively short SOA not allowing for the power to return to baseline before the next stimulus was presented. Furthermore, the SOA was fixed making entrainment and anticipation possible. These were deliberate design choices to be able to collect a large number of trials for model-based analyses. Any jittering of the SOAs (which would have to go in the direction of increasing their duration) would have led to far fewer trials being collected. The total duration of the recording had to be kept short as the patients were unable to tolerate extended periods of testing. Furthermore, having a very long SOA would make it more likely that the participants would resort to explicit counting, which was something we aimed to avoid.

To account for these design issues, we developed an unconventional way of performing time-frequency analysis on these data in the absence of a baseline. We first ran time frequency analysis on continuous LFP data [multitaper method (Thomson, 1982) 400-ms sliding window, in steps of 50 ms] on a priori defined β power (13–30 Hz average = 21.5 Hz; note that when looking at individual participant β power around the response period, we found a similar band as defined *a priori*: individual mean range: 16.6–28.4 Hz; overall min: 11 Hz, max: 31 Hz). Separately we also estimated the power in the θ band (2–8 Hz average = 5 Hz; Herz et al., 2016). The resulting power time series were log-transformed and high-pass filtered at 0.5 Hz (Butterworth fifth order, zero phase filter) to remove fluctuations in power that were slower than our SOA. Afterwards, the power time series were epoched around the presentation of each cue stimulus (−500–800 ms). We averaged power across contacts within each hemisphere, resulting in 1 left and 1 right STN channel, and we also calculated the mean STN signal by combining hemispheres. We used a permutation cluster-based non-parametric test to correct for multiple comparisons across time (the duration of the whole cue epoch (0–800 ms) and report effects that survive correction only [*p* < 0.05 family-wise error (FWE) corrected at the cluster level].

Similarly to LFP, MEG data were downsampled to 300 Hz, and high-pass filtered at 1 Hz (Butterworth fifth order, zero phase filter). For sensor-level analysis, we used only the control group data, as in the patients the sensor signals were contaminated by ferromagnetic wire artefacts (Litvak et al., 2010).

For the MEG sensor-level time-frequency analysis, we used all channels and a frequency range of 1–45 Hz. All other analyses were identical to the LFP pipeline reported above except we corrected for multiple comparisons across all MEG channels, timepoints (0–800 ms) and frequencies (1–45 Hz), and only report effects that survived that correction (*p* < 0.05 FWE corrected at the cluster level).

For source-level analysis, the continuous MEG data were projected to source space with linearly constrained maximum variance (LCMV) beamformer (Veen et al., 1997) using a 10-fold reduced version of the SPM canonical cortical mesh (Mattout et al., 2007) as the source space (resulting in 818 vertices and the same number of source channels). The source orientation was set in the direction of maximum power. See Litvak et al. (2012) for details on beamforming and Litvak et al. (2010) for details on issues regarding beamformer use for removing artefacts from simultaneous MEG and intracranial recordings. Next, time-frequency analysis was performed on continuous source data the same way as for STN-LFP except the frequencies of interest were informed by the sensor-level analysis. This biased the statistical test for discovery of an effect (cf. double dipping; Kriegeskorte et al., 2009) but our aim in this analysis was *post hoc* interrogation of the effects established at the sensor level in terms of their location in the cortex rather than hypothesis testing (Gross et al., 2013). To limit our search space for the coherence analysis (below), we only investigated sources that survived *p* < 0.05 FWE correction.

Time-resolved coherence was then computed between the identified cortical sources and STN contacts by going back to raw source time series. The data were epoched (−1000–1000 ms to increase the window for analysis), and time-frequency analysis was performed as described above with coherence between the sources and the left and right STN also computed from the cross-spectrum. Non-parametric permutation testing between conditions was corrected for multiple comparisons across channels (source vertices), time (0–1600 ms to cover both cue “i” and cue “i + 1”), and frequencies (1–30 Hz), and we only report effects that survive correction (*p* < 0.05 FWE corrected at the cluster level). For completeness, we also ran an alternative connectivity measure, debiased weighted phase lag index, which is less sensitive to unequal trial numbers across conditions and volume conduction effects.

### Reconstruction of electrode locations

We used the Lead-DBS toolbox (http://www.lead-dbs.org/; Horn and Kühn, 2015) to reconstruct the contact locations. Postoperative T2 and T1 images were co-registered to preoperative T1 scan using linear registration in SPM12 (Friston et al., 2007). Preoperative (and postoperative) acquisitions were spatially normalized into MNI_ICBM_2009b_NLIN_ASYM space based on preoperative T1 using the Unified Segmentation Approach as implemented in SPM12 (Ashburner and Friston, 2005). DBS electrode localizations were corrected for brain shift in postoperative acquisitions by applying a refined affine transform calculated between preoperative and postoperative acquisitions that was restricted to a subcortical area of interest as implemented in the brain shift correction module of Lead-DBS software. The electrodes were then manually localized based on postoperative acquisitions using a tool in Lead-DBS specifically designed for this task. The resulting locations were verified by an expert neurosurgeon.

### Choice strategy

In order to analyze the strategy used by the participants during choice, we investigated which factors influence commitment to a choice on a given trial. We considered two factors: the first of them is the evidence integrated for the chosen option. Such accumulated evidence was computed from Equation 1 that continuously updates the evidence (decision variable, *DV*) for a choice at time *t* based on the existing *DV* from the previous stimuli and the new incoming stimulus $S_{t}$, where $S_{t}=-1$ for the left stimulus, and $S_{t}=1$ for the right stimulus. At the start of each trial, the *DV* was initialized to $DV_{0}=0$.

(1) $$DV_{t}=DV_{t-1}+S_{t}.$$

The second factor we considered was whether the stimulus was the same as the previously presented one, i.e., $SA_{t}=1$ if $S_{t}=S_{t-1}$ and $SA_{t}=0$ otherwise. For all stimuli excluding the first stimulus on each trial (for which it is not possible to define $SA_{t}$) we performed a logistic regression predicting if the choice has been made after this stimulus, i.e., we tried to predict a variable $D_{t}=1$ if choice made after stimulus *t* and $D_{t}=0$ otherwise. For each participant, we looked at the significance of the two factors.

### Estimating accumulated evidence using computational models

In order to analyze whether STN activity reflects the amount of available evidence for each response based on the stimuli presented so far, we employed computational models that can estimate this quantity at each point in time. We compared how well different models of evidence accumulation could capture the behavior of different patients, and then generated regressors for each patient based on the best model for that patient. In addition to the model assuming evidence is integrated according to Equation 1, we also considered three extended models which included a forgetting term (λ), a bonus term (ω), or both (Eqs. 2–4).

(2) $$DV_{t}=\left(1-\lambda \right)DV_{t-1}+S_{t}$$

(3) $$DV_{t}=DV_{t-1}+\left(1+\omega SA_{t}\right)S_{t}$$

(4) $$DV_{t}=\left(1-\lambda \right)DV_{t-1}+\left(1+\omega SA_{t}\right)S_{t}.$$

The forgetting term was used to model the decay of memory over the course of the trial and the bonus term is a weighting of “same” pairs, i.e., the stimuli which match the directly preceding one (e.g., in a “left-left-right” sequence the second left stimulus would be weighted extra as it is the same as the first one).

To estimate the parameters (λ,ω), we assumed that the ratio of making a right choice to making a left choice is related to *DV* according to:

$$\text{ln}\frac{P\left(R\right)}{P\left(L\right)}=\beta_{0}+\beta_{t}DV_{t}.$$

For each participant, we looked for parameters that maximized the likelihood of participant's behavior after all stimuli shown to that participant.

We found the winning model (based on Bayesian information criterion) to be variable across participants (number of participants in patients/control group indicated): M1 = 1/2; M2 = 0/0; M3 = 4/9; M4 = 8/2, although the models that included bonus terms were most common.

### Estimating Bayesian normalization term

We investigated whether the STN activity follows a pattern predicted by a computational model of the basal ganglia (Bogacz and Gurney, 2007; Bogacz and Larsen, 2011). This model suggests that the basal ganglia compute the reward probabilities for selecting different actions according to Bayesian decision theory. These probabilities are updated after each stimulus and the updated information is fed back to the cortex via the thalamus. An action is initiated when the expected reward under a particular action exceeds a certain threshold. The model attributes a very specific function to the STN: ensuring that if the probability of one action goes up, the probabilities of the others go down at the same time by normalizing all probabilities so that they add up to one.

In order to create regressors for neural activity recorded from the STN, we used the original proposal that the STN computes the normalization term of the Bayesian equation during the evidence integration process (Bogacz and Gurney, 2007). We defined two cortical integrators *Y_L_* and *Y_R_*, which integrate evidence for the left and right stimulus respectively, as described above. Additionally, we subtracted the STN normalization term from the cortical integrators after each stimulus input in a sequence (Bogacz et al., 2016). For each participant, we assumed the integration follows one of the models described by Equations 1–4, which best describes given participants (see above, Estimating accumulated evidence using computational models). So, for example, for participants best described by Equation 1, the integrators were updated as follows:

(5) $$Y_{L,t}=Y_{L,t-1}+L_{t}-STN_{t-1}$$

(6) $$Y_{R,t}=Y_{R,t-1}+R_{t}-STN_{t-1}$$

(7) $$STN_{t}=\text{ln}\left(expY_{L,t}+expY_{R,t}\right).$$

**Figure 2.**
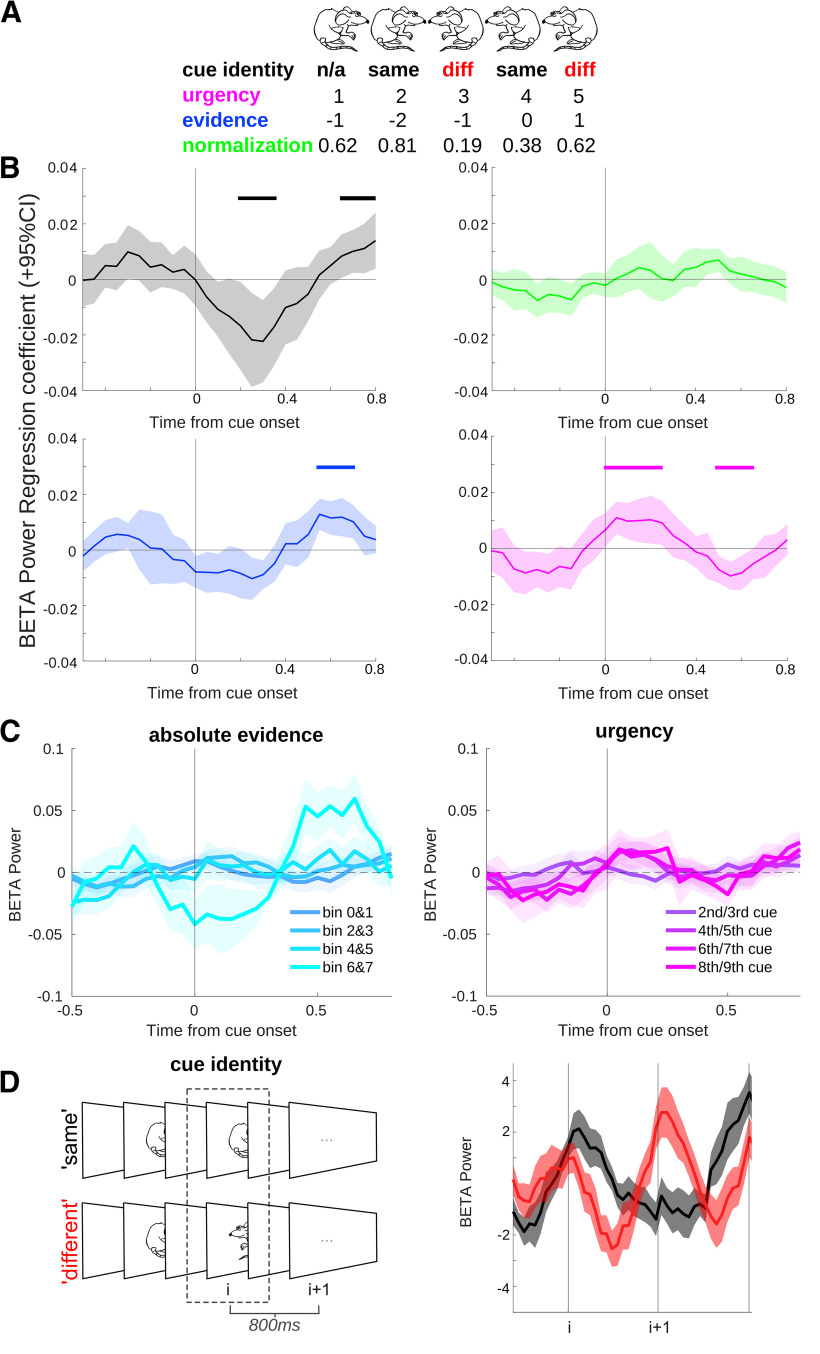
STN activity encodes local conflict and variables related to accumulation of evidence via β oscillations. ***A***, Example sequence of cues, with each regressor value shown below. For example, evidence for the “right” facing mouse goes up during the first two cues, but then the appearance of a “left” mouse reduces the evidence for a right response. ***B***, Results of the combined General Linear Model (GLM). A linear regression of β power in the STN revealed that a clear signal was related to the identity of the cue (“same” or “different,” shaded in gray), absolute integrated evidence, and sample number in the sequence of cues in a trial (or “urgency,” i.e., the number of stimuli presented so far that could influence a general tendency to make a choice or working-memory load). Horizontal lines represent significant times after cluster correction for multiple comparisons. There was no encoding of Bayesian normalization in the STN signal, as proposed previously (Bogacz and Gurney, 2007; Bogacz et al., 2016). Note that although the regressors are presented separately for easier visualization, they were included in a combined GLM. All regressors were z-scored before entering the model. We did not find any effects when regressing θ band activity in the STN with the above regressors. ***C***, Raw β power plotted as a function of binned evidence (left) or cue number (right), as well as for cue identity (***D***), note this latter panel is identical to part of Figure 3*B*. See Extended Data Table 2-1 and Extended Data Figure 2-1 for correlations performed to relate neural effects to behavior.

In the above equations, $L_{t}=1$, $R_{t}=0$ if cue t is left, and $L_{t}=0$, $R_{t}=1$, otherwise. However, for models 2–4, we added decay to the cortical integrators and bonus terms to Equations 5, 6 analogously to Equations 2–4, i.e., we ensured that $DV_{t}=Y_{R,t}-Y_{L,t}$. At the start of each trial, the integrators were initialized to $Y_{L,0}=Y_{R,0}=\text{ln}0.5$ (corresponding to equal prior probabilities of the two responses). The value computed from Equation 7 was used as Bayesian normalization regressor in Figure 2.

### Data availability

The full MEG dataset for controls is available in BIDS format on https://openneuro.org/datasets/ds002908 and LFP and source data for patients is available on https://data.mrc.ox.ac.uk/data-set/human-lfp-recordings-stn-during-sequential-conflict-task. Code and analysis pipeline at https://github.com/zits69/MOUSE_LFPMEG.

## Results

### Patients are able to accumulate evidence over time

Patients waited on average 6.6 stimuli before making a response (6.59 ± 0.52 SEM), and their accuracy was significantly above the 70% level expected if they only based their decision on a single cue (80 ± 0.03 SEM, *t* = 3.6, *p* = 0.004). Controls waited on average 6.3 stimuli before making a response (6.29 ± 0.46 SEM) and were similarly above 70% in their accuracy (88.6 ± 0.01 SEM, *t* = 18.4, *p* < 0.001). There was no significant difference between groups in the number of stimuli viewed before making a choice (*t* = 0.42, *p* = 0.68), but patients had lower accuracy (*t* = −2.99, *p* = 0.0009) and slower reaction time (RT, as measured from the onset of the last cue before a response was made, *t* = 2.16, *p* = 0.041). See Table 1 for summary of behavioral measures.

**Table 1.** Behavioral results showing mean and SDs for each group

	# Stimuli seen	Accuracy	RT (ms)	Fraction of responses after “same” at end
Patients mean	6.59	0.80	536.52	0.73
Patients SD	1.88	0.10	29.48	0.11
Controls mean	6.29	0.89	502.74	0.81
Controls SD	1.65	0.04	48.81	0.09

RT, reaction time; SD, standard deviation. The analytical probability of a “same” pair at the end of the sequence would be 58% if participants chose the moment of response randomly. Both patients and controls responded significantly more often after a “same” pair (both groups *p* < 0.001).

To explore potential strategies participants could have used in the task, we compared performance in both groups to an agent that would have been an optimal observer, and would choose to respond left if the number of left cues was higher than the number of right cues, to respond right for a larger number of right cues, and would choose randomly if the numbers were equal. In other words, for each participant, we calculated the accuracy they would have achieved had they integrated evidence optimally, having seen the stimuli sampled by the participant on each trial. We found that controls and patients had significantly lower accuracy (controls: *p* = 0.019, patients: *p* = 0.0076) than an ideal observer would have, based on the same cue sampling (89% for controls and 87% for patients).

Next, we asked whether participants were just solving the task by responding after they spotted two of the same stimuli in a row (i.e., after the first “same” pair). To address this question, we investigated to what extent participants' response after stimulus was predicted by accumulated evidence, and by same stimuli in a row (for details, see Materials and Methods). Most participants had responses best predicted either by accumulated evidence alone (six patients and six controls), or by both accumulated evidence and stimulus repetition (five patients and seven controls). For remaining two patients none of these factors was predicting their response. Hence, there was no participant who exclusively relied of making a choice after seeing the “same” stimulus, without considering evidence integrated so far.

### STN β power reflects multiple variables related to ongoing decision-making

In order to understand the impact of different variables related to the decision-making process on activity in the STN, we created a combined General Linear Model (GLM), including four regressors: cue identity, normalization model, accumulated evidence, and sample number. These are described in detail below.

Cue identity was taken as a measure of “local conflict,” by taking all cues (excluding the first and last cues in a sequence) and categorizing them as the “same” or “different” from the previous cue (Fig. 2*A*,*D*). We found that β power carried information about the similarity of the stimulus to the previous one (“cue identity,” 200–350 and 650–800 ms, *p* = 0.024 and *p* = 0.032; see Fig. 2*B*,*D*).

10.1523/JNEUROSCI.0572-21.2022.tab2-1 Extended Data Table 2-1 Correlations between β power (different – same), model regressors and behavioral measures. We correlated across participants the changes in β power at each cue (cue “i,” “i + 1”) with behavioral measures (accuracy, reaction time, the number of stimuli sampled, proportion of trials ending on a “same” cue). When correlating trial-wise β power with reaction time or the number of stimuli sampled at the single participant level, we did not find any significant effects. Other than raw power changes, we also included the full GLM regression values from Figure 2 as well as the coherence effects from Figure 5. Note, the listed *p*-values are uncorrected, and thus the two correlations with *p* < 0.05 would not survive the correction for multiple comparisons. *If outlier is taken out then correlation is no longer significant (*r* = 0.47, *p* = 0.12), see Extended Data Figure 2-1 for reaction time (RT). Outlier detected as more than 1.5 interquartile range above the upper quartile or below the lower quartile, which is appropriate when data is not normally distributed. Download Table 2-1, DOCX file.

10.1523/JNEUROSCI.0572-21.2022.f2-1 Extended Data Figure 2-1 Correlation between cue identity regressor and reaction time, and between coherence and number of cues sampled Note the *p*-values associated with these correlations do not survive correction for multiple comparisons. Download Figure 2-1, TIF file.

In addition to local conflict, we analyzed whether other variables occurring in theoretical models of decision-making were reflected in neural activity. We explored whether STN represents the normalization term in Bayes theorem as proposed in a previously suggested computational model (Bogacz and Gurney, 2007). This model predicts that the activity in the STN is proportional to a logarithm of the normalization term in Bayes theorem ln P(cue i). This probability is computed on the basis of all previous cues {cue 1, …, cue i – 1} so it expresses how expected the current cue is given all cues seen before. The negative of this regressor, -ln P(cue i), is equal to Shannon's surprise, so it expresses how much cue i disagrees with overall information in all previous cues, and hence it could be viewed as a measure of global conflict. Therefore, a possible correlation between the normalization term ln P(cue i) and LFP activity could be explained by either of two hypotheses. A computational model (Bogacz and Gurney, 2007) predicts a positive correlation, whereas a hypothesis that STN responds to global conflict predicts a negative correlation. We tested whether the normalization term affects power of β oscillations in the STN and did not find evidence supporting any of these two hypotheses in our data (Fig. 2*B*).

We also explored whether there was a signal reflecting the magnitude of accumulated evidence in the STN, observed in a similar task (Gould et al., 2012). Additionally, we included a regressor on β power equal to the serial position of the cue stimulus within a trial. Including this regressor was motivated by two observations: reports of decreasing β power as a result of increasing working memory load (Zavala et al., 2017), and presence of “urgency signals” in the basal ganglia that increase within a trial and reflect the growing urgency to making a choice (Thura and Cisek, 2017). We found a significant effect in both regressors (absolute evidence: 550–700 ms, *p* = 0.008; cue number or urgency: 0–250 and 500–650 ms, *p* = 0.01 and *p* = 0.02).

We did not find a clear relationship between behavior on the task and these neural effects (see Extended Data Table 2-1). However, cue identity (early peak) showed a relationship with both RT (*r* = 0.62, *p* = 0.024; note that if an outlier of the STN data is taken out, then the correlation is no longer significant, *p* = 0.12; outlier detected as >1.5 interquartile range above the upper quartile or below the lower quartile, which is appropriate when data are not normally distributed), as well as a trend for the number of cues sampled (*r* = 0.53, *p* = 0.064).

### STN β power shows persistent activity to local conflict during evidence accumulation

**Figure 3.**
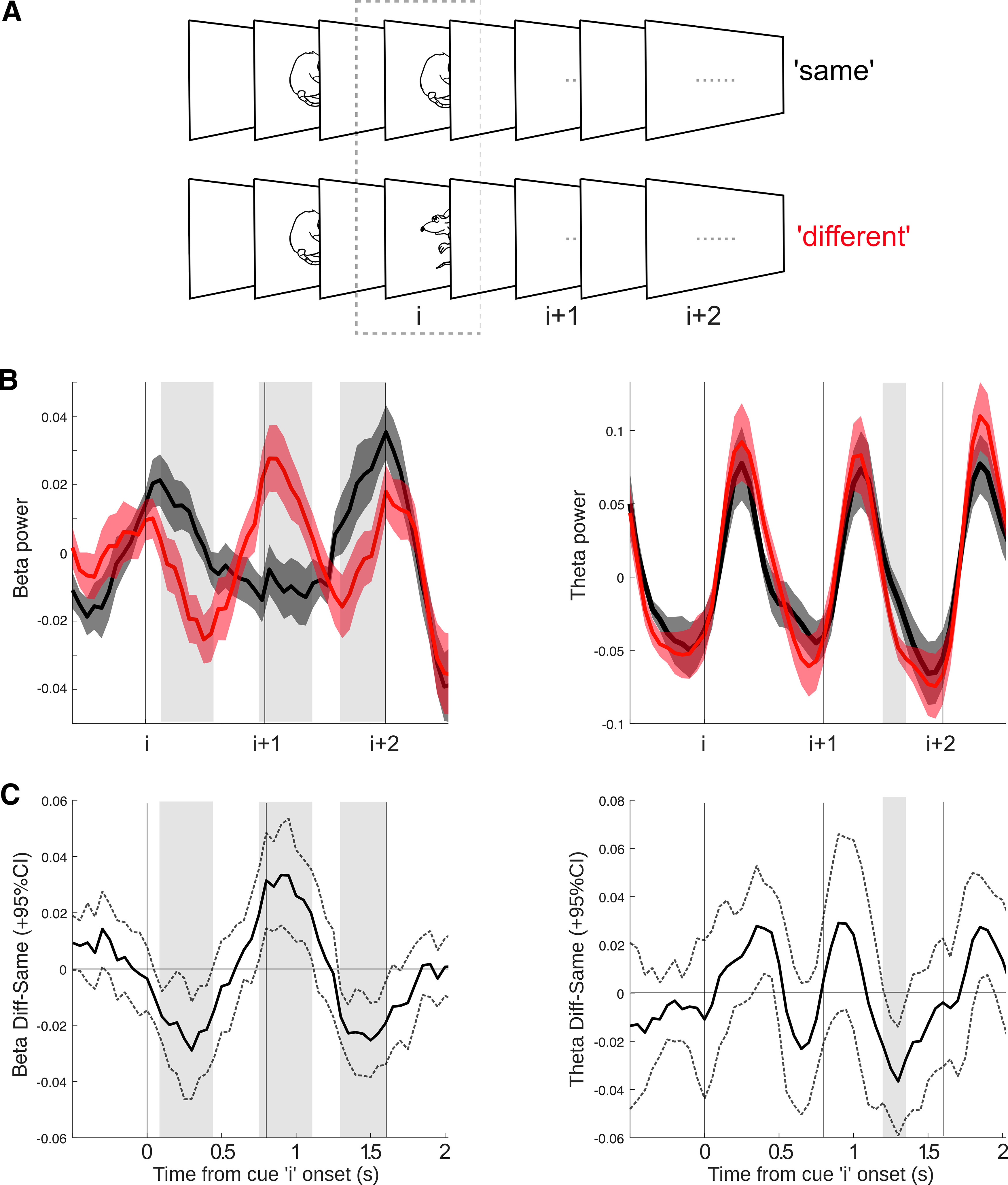
β signaled local conflict, and carried this effect over to the next cue in a sequence. ***A***, Notation used in the paper. Let us consider an arbitrary cue i in a sequence, where i > 1: If cue i – 1 is the same as cue i, then we would call this the “same” condition, and “different” otherwise. We also plot the subsequent cues (i + 1, i + 2) for carry-over effects, but these are collapsed across cue type, left or right. See Extended Data Figure 3-1 for more details. ***B***, Left panel, β carried information locally as well as over to the next cue, with increased β power for the “different” condition. Right panel, θ only carried mismatch information at the next cue in the sequence. Significant time periods are highlighted with shaded gray bars. Vertical lines show onset of cues in the sequence. The shaded error bars show standard error of the mean. ***C***, Difference waves of conditions (“different” minus “same”) with 95% confidence intervals (CI) shown by the dotted lines. After an initial dip there is a clear increase in β power following the conflicting cue (i) starting just before the onset of cue i + 1. Significant time periods are highlighted with shaded gray bars copied from panel ***B*** for comparison. Note that the apparent onset of the effect before zero is because of limited time resolution of the time-frequency decomposition.

Complementing, and extending on the above regression analyses, to further investigate how the STN represents the inconsistencies when faced with conflicting evidence over time, we separated all cues into two categories: “same” or “different” to the one immediately before it (we term this “cue i”; Fig. 3*A*). In our analyses of neural responses to cues, we excluded the first cues in a sequence, because it is not possible to classify them as “same” or “different,” and last cues seen as they overlapped with the response period. Thus, if a participant experienced this sequence of mouse images: “left-right-left-left-right,” the analyzed conditions would be “different-different-same.”

10.1523/JNEUROSCI.0572-21.2022.f3-1 Extended Data Figure 3-1 Effects from Figure 3, plotted with cue i + 1 in detail; for example, “same”-“different” could be a cue sequence: “L-L-R.” Plotted is the response to the last cue of the triplet, “R,” in this example. Top, β Power. Bottom, θ Power. Download Figure 3-1, TIF file.

We found that β oscillations (i.e., raw β power) responded to local conflict, generating a significant difference between “same” and “different” cues (cue “i” in Fig. 3*B*, left panel) starting around 100 ms after cue onset. β also showed a significant difference in the subsequent cue (i + 1), with “different” cues showing an increase in β power, thus conflicting information on cue i results in increased β power on cue i + 1 (see Fig. 3*C*), a pattern of activity that is consistent with response inhibition. Significant time clusters: 100–450 ms (*p* = 0.022, *d* = 1.74), 750–1100 ms (*p* = 0.014, *d* = 1.73), 1300–1600 ms (*p* = 0.012, *d* = 2.40). These effects were greatly reduced in the θ band, with an effect of condition only briefly detectable during cue “i + 1” (Fig. 3*B*,*C*, right panel).

### Cortical activity reflects rapid but nonpersistent local conflict detection

**Figure 4.**
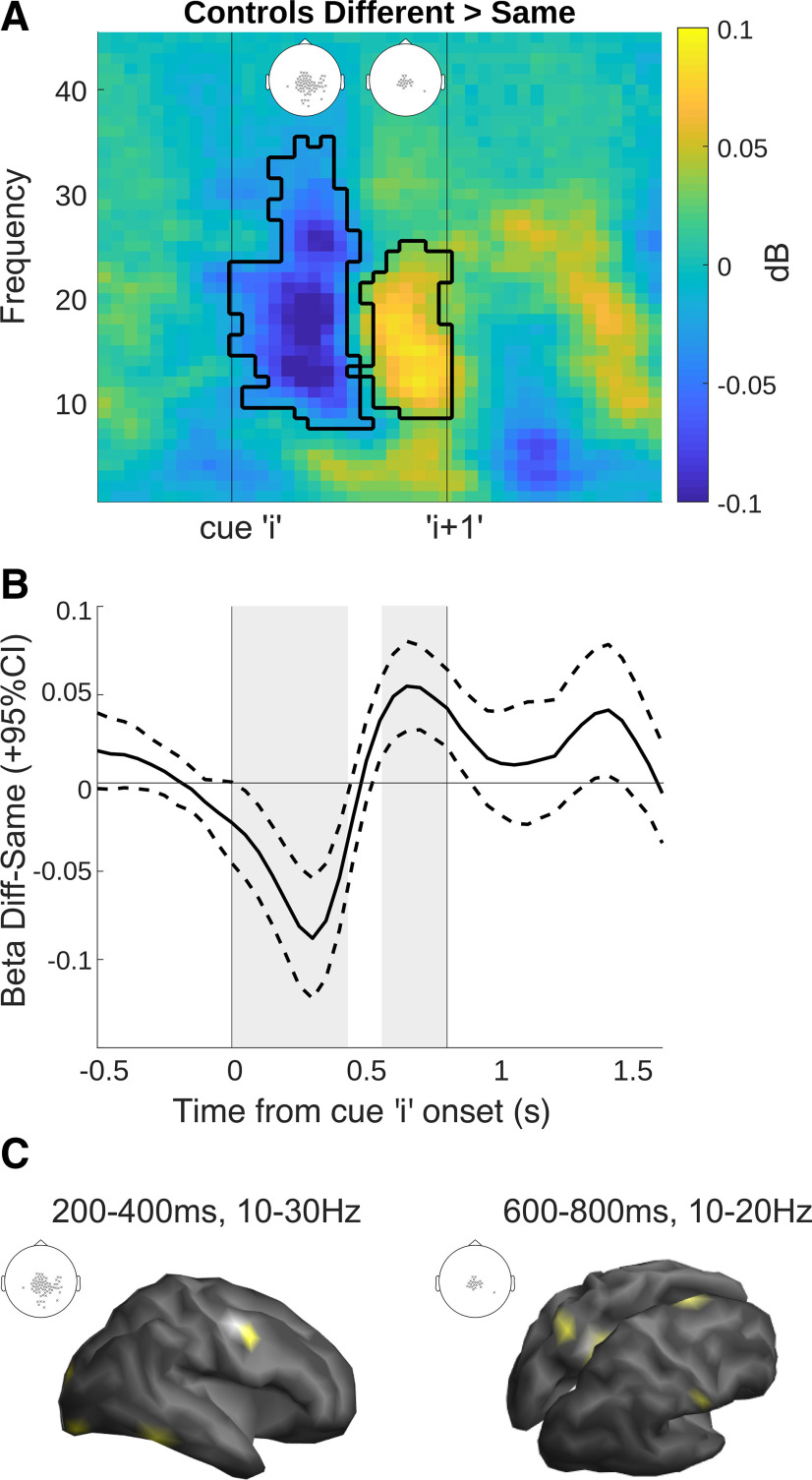
Cortical activity to local conflict parallels STN but peaks earlier on average and has a shorter time course. ***A***, Time-frequency plot showing significant times and frequencies when contrasting “different” versus “same” cues, averaged over all significant sensors. Significant sensors are shown as an inset, separately for the two clusters (cluster 1: 0–450 ms, 8–35 Hz; cluster 2: 550–800 ms, 9–25 Hz). ***B***, Difference wave for the β effects over clusters (13–30 Hz) band, as represented in Figure 3*B*. The dotted lines indicate 95% confidence intervals (CI). ***C***, Left, Source localization in a combined sample of patients and controls revealed the source of cluster 1 in three right-lateralized areas: occipital pole, ventral temporal cortex and lateral premotor cortex (BA6). Right, Cluster 2 showed left lateralized superior parietal lobe (BA7), left posterior cingulate cortex (BA23), right primary sensory cortex and right dorsal premotor cortex/presupplementary motor area (dPM/BA6).

We investigated sensor-level MEG signals from controls in response to local conflict detection within the sequence. As with the STN, widespread activity over central sensors was found to signal local conflict, with an initial dip followed by an increase in β power on “different” trials (Fig. 4*A*). The dip and increase in β power were associated with different clusters of electrodes. The first cluster showed a significant decrease to different cues in the β band across central, and predominantly right occipital, parietal and temporal sensors (Fig. 4*A*, inset; 0–450 ms, 8–35 Hz, *p* = 0.002, Cohen's *d* = 1.22). A subsequent second cluster, more restricted to central sensors, showed an increase in β power to different cues (550–800 ms, 9–25 Hz, *p* = 0.008, Cohen's *d* = 1.35).

Interestingly, the time course of the cortical effect was quicker than that of the STN (Figs. 4*B* vs 3*B*), with conflicting information only lasting until the onset of the next cue in the sequence.

### Coherence is increased between STN and frontal cortex during local conflict

We used beamforming in a combined sample of patients and controls to localize the source of the “same-different” effect [cluster 1: averaged over: 200–400 ms (to exclude the time the stimulus was displayed on the screen), 10–30 Hz; cluster 2: averaged over 600–800 ms, 10–20 Hz]. In cluster 1, we found three right-hemisphere lateralized peaks (Fig. 4*C*): occipital pole (two peaks: Montreal Neurological Institute (MNI) coordinates 19, −98, −14; 35, −89, −16), ventral temporal cortex (two peaks: MNI 59, −53, −21; 52, −51, −21), and lateral premotor cortex (Brodmann Area (BA) 6, two peaks: MNI 52, −7, 44; 51, 3, 40). Cluster 2 was localized to left superior parietal lobe (SPL/BA7, MNI −23, −61, 52), left posterior cingulate cortex (PCC/BA23, MNI −14, −47, 31), right dorsal premotor area (dorsal/medial BA6, MNI 7, 2, 69), and right primary somatosensory cortex (BA1, MNI 61, −18, 31). Note, at an uncorrected threshold (*p* < 0.001) we also found the lateral premotor cortex, occipital pole and temporal cortex as in cluster 1, which is expected given the overlapping topography of sensors in the two clusters.

**Figure 5.**
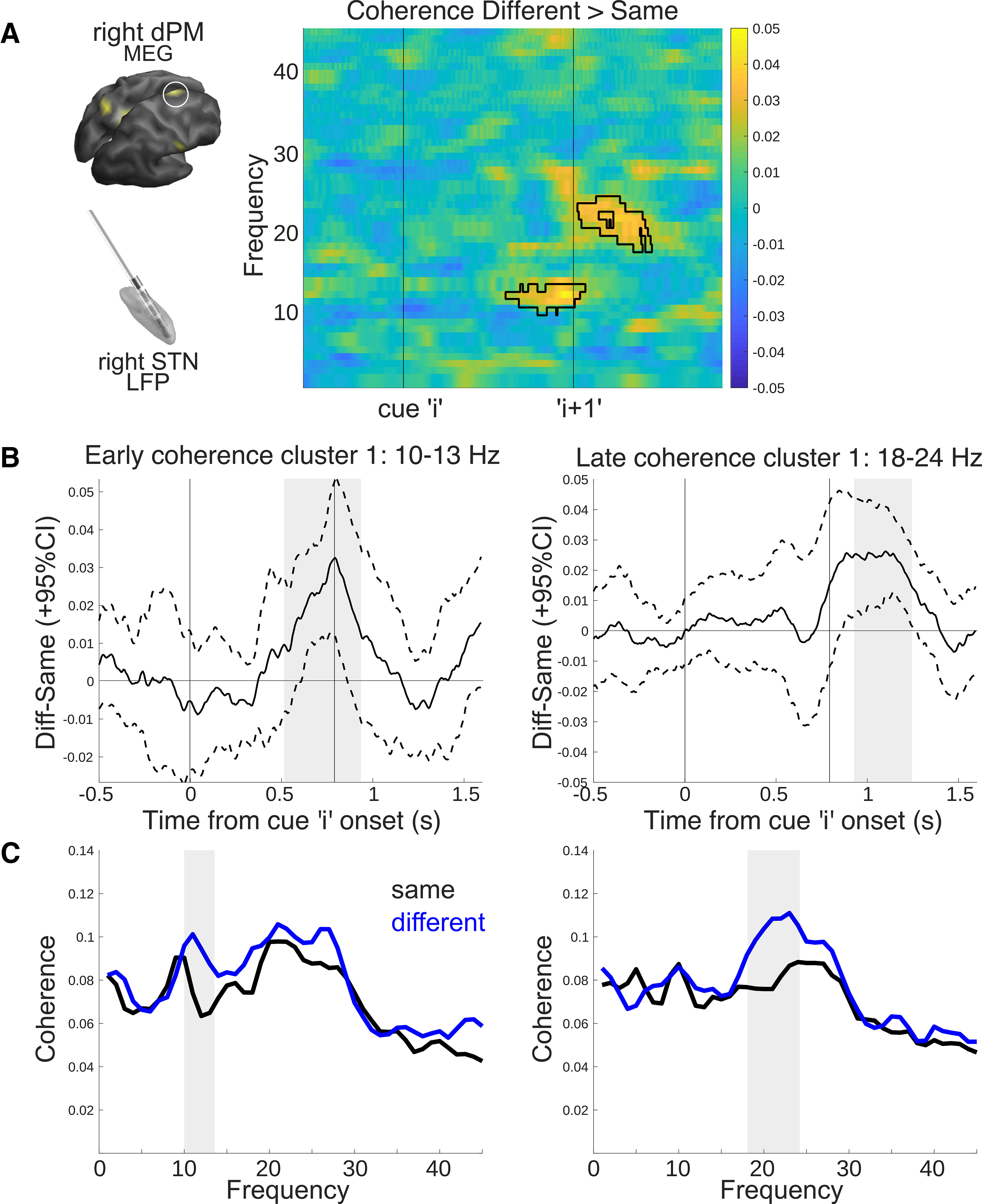
Increased coherence between right frontal cortex and right STN during local conflict. ***A***, Time-frequency plot of coherence between the right STN and the right dorsal premotor cortex (visualized on the left). Two coherent clusters emerged, with an α/low β coherence increase after “different” cues, and a later increase in β coherence carrying over into the next cue in the sequence. Significant clusters are shown in black outline. Inset on top left shows the source of the cortical effect for reference. ***B***, Time courses of coherence for both α/low and high β plotted as a difference wave between conditions. The dotted lines indicate 95% confidence intervals (CI). Significant timepoints are highlighted in gray. ***C***, Frequency spectra of “same” (black) and “different” (blue) trials during the significant time period from ***A***. Gray area highlights significant frequencies:10–13, 18–24 Hz.

Next, we measured in patients the coherence between these cortical vertices and both the left and right STN-LFPs, separately. The coherence spectra were averaged over adjacent vertices resulting in three cortical sources for cluster 1 and four sources for cluster 2. We found a significant increase in coherence between the right dorsal premotor cortex and the right STN (510–900 ms, 10–13 Hz, *p* = 0.03, Cohen's *d* = 1.71; 900–1240 ms, 18–24 Hz, *p* = 0.01, Cohen's *d* = 1.44; see Fig. 5), suggesting that ipsilateral cortico-subthalamic coherence is increased in the face of local conflict in the right hemisphere. Furthermore, it seems there are two separate points of coherence over the course of the cue, one after the onset of the conflict cue and one that extends into the processing of the next cue in the sequence, this latter effect is in the mid-high β band, possibly reflecting response inhibition. No other sources, nor the left STN showed any significant effects. For completeness based on previous reports, we also investigated coherence with the inferior frontal gyrus (IFG; which was present as a source in patients at an uncorrected threshold), and found that it did not show any significant coherence with the STN. We also used debiased weighted phase lag index as an alternative measure and found the same effects, albeit with reduced significance (cluster 1: 690–910 ms,10–13 Hz, *p* = 0.043; cluster 2: 860–1150 ms, 20–24 Hz *p* = 0.056).

## Discussion

In this experiment, we present novel evidence pertaining to the role of the STN and cortico-subthalamic communication during sequential decision-making, using a task in which participants had to integrate evidence over discrete time periods, with no constraints on how many samples they could observe before making a decision. We find evidence for persistent local conflict representation in the STN via β oscillations, and increased coherence with frontal cortex. We also observed modulation of β power in STN by evidence accumulation and number of cues presented so far in a trial.

### Representation of conflict in the STN

We found that activity in the β band carried information about local conflict, i.e., a difference between the current cue and the preceding one, but not about global conflict i.e., a surprise by the current cue given all previous cues. Although we established that β power varies depending on whether the current cue differs from a previous one in a sequence, an event to which we refer as a local conflict, it is less clear from our data what the function of this activity is, and what fundamental variable it encodes.

It is possible that the observed changes in β power are connected with motor inhibition. β power was initially lower for cues that were “different” to the one immediately before and continued to increase across the next cue in the sequence. Activity in the β band has been shown to carry conflict information across trials (Zavala et al., 2018), but we also show this effect within a trial, as conflict arises within the sequence of evidence. Hence, one can interpret the increase of β power as a stop signal, or a break on motor output (Alegre et al., 2013) inhibiting a response after an inconsistent cue. Moreover, the majority of trials ended on a “same” cue (Table 1), which is in line with an overall increase in β synchronization after “different” cues and lower probability of responding.

The response to different cues could also be interpreted as encoding of expectancy valuation, uncertainty or surprise. β Power increases have been reported when a “surprise” stimulus is presented (Wessel et al., 2016), and STN activity measured with fMRI has been shown to increase when there is increased uncertainty which option is correct arising because of too much choice (Keuken et al., 2015). However, in our study we found no evidence that the STN encodes the Shannon's surprise term.

### Interaction between STN and cortex

Interestingly, the “same”-“different” effect on average peaked earlier in the cortex, and also did not carry over to the next cue in the sequence (Fig. 4*A*). A possible interpretation is that the cortex signaled the immediate local conflict to STN, dovetailing with recent evidence suggesting the cortical conflict signal precedes the STN (Chen et al., 2020), which then maintains a more persistent activity to inhibit responses (Brittain et al., 2012; Fife et al., 2017).

When we localized the sources of the “same”-“different” effect, we found the local conflict signal in widespread areas of the cortex. Only one frontal source, located in dorsal premotor cortex/supplementary motor area (dPM/BA6) showed a significant coherence modulation with the ipsilateral STN only, namely an increase in α/low-β coherence shortly after the offset of a “different,” or conflict, cue, and an increase in β coherence that carried over to the next cue in the sequence (Fig. 5). The right BA6, specifically dorsal BA6 (Mattia et al., 2012; Mirabella, 2014), is well established as a cortical region involved in response-inhibition/initiation and cognitive control (Chambers et al., 2007; Simmonds et al., 2008; Aron, 2011).

While it is well established that the cortex communicates with the STN via two anatomically defined pathways, the indirect and the hyperdirect pathways (Albin et al., 1989; DeLong, 1990; Nambu et al., 2002), recent evidence suggests the existence of two separate coherent β oscillatory networks between the cortex and the STN (Oswal et al., 2016a). Here, we find evidence for two different bands of oscillatory connectivity between the STN and dorsal premotor cortex, which may have implications for understanding the involvement of various pathways in sequential evidence accumulation. Interestingly, a recent study showed evidence of a hyperdirect pathway from IFG to the STN operating in the 13- to 30-Hz range (Chen et al., 2020), which points to a more ventral portion of the frontal cortex than presented here. In fact, many studies in stop-signal/go-no-go tasks point to the IFG (Aron et al., 2014); however, in these tasks, conflict is not part of an evidence accumulation process, hence we may expect differences depending on the type of decision being made (Erika-Florence et al., 2014; Hampshire, 2015; Mosley et al., 2020).

Because of the evoked-activity as a result of the ongoing cue presentation, we were unable to reliably estimate the directionality of coherence, but previous reports on resting-state data have shown cortex to drive STN activity (Litvak et al., 2011a), which is in line with the finding here that the “same”-“different” effect seems to peak earlier in the cortical signal. However, recent data has also suggested that during processing of incongruent stimuli, STN to primary motor effective connectivity is increased in the β band (Wessel et al., 2019), suggesting that the directionality of communication may be different across task and non-task contexts.

### Where is the θ conflict signal?

The predominant theory of STN function, and also that of the cortex during conflict detection, is the involvement of θ oscillations (Cavanagh and Frank, 2014). A large portion of empirical findings on the STN shows that it carries conflict information via the θ band (Cavanagh et al., 2011; Bastin et al., 2014; Zavala et al., 2015, 2016, 2017, 2018; Herz et al., 2016). Yet in our task we only found a weak effect of θ modulation, in the cue following a local conflict (cue i + 1). This effect was present only in the STN, and no θ effects were found in the cortex. Moreover, this manifested as reduced θ synchronization to “different” cues, which is the opposite of the standard reported θ increase during conflict. One explanation may be the task design, as it differs from previous paradigms: there are no long intervals over which to examine slow oscillations, such as θ. Our results, therefore, though focused on θ power, may be dominated by evoked potentials, as cues were presented in a fixed, relatively short duration sequence. Additionally, here conflict is defined over the course of multiple cues, not on a singular trial in isolation. Thus, the integration of conflict over time may in fact be driven by different signals, β may represent a more consistent inhibition. Nevertheless, others have also reported a lack of θ effects in the STN during a stop-signal task (Bastin et al., 2014).

### Updating models of the STN

An influential model of the role of the STN in decision-making proposed by Frank (2006) suggests that in situations of conflict between competing responses an increased activity of STN postpones action initiation (Frank, 2006). This model proposes that STN is essential for decision-making since it ensures that an action is only selected when it has high evidence, relative to the other options. Another model proposed by Bogacz and Gurney (2007) suggests that the basal ganglia compute the reward probabilities for selecting different actions according to Bayesian decision theory (Bogacz and Gurney, 2007; Bogacz and Larsen, 2011). While in our task we did not find conclusive evidence that the STN is encoding Bayesian normalization (Fig. 2*B*), it is important to remember that, despite being on medication, these experiments were performed in patients whose neural circuitry has been affected by advanced Parkinson's disease. Thus, one cannot rule out the possibility that the Bayesian normalization is encoded by the STN of healthy individuals, but testing this hypothesis would require a different experimental technique (e.g., recording of STN neural activity from animals during an analogous decision-making task, such as in Brunton et al., 2013). Evidence also suggests that subdivisions within the STN may be responsible for different types of inhibition, with prepotent response inhibition to cues (go-no-go task) being more dependent on the ventral portion of the STN (Hershey et al., 2010). Given that the majority of our recording sites were well within the dorsal (“motor”) region of the STN, we cannot rule out the contribution of more ventral sites to these computations.

We conclude that contrary to the emphasis on θ signals in the context of immediate conflict, here we find a prominent role for β oscillations in signaling local conflict in a sequence of evidence. We find that both frontal cortex and the STN carry this signal, and show increased coherence in the β band that carries over to the next cue in the sequence. Thus, we show increased communication in these areas may reduce the probability of responding in the face of incoming conflicting information.
