# Supplementary material for: Conflict Detection in a Sequential Decision Task Is Associated with Increased Cortico-Subthalamic Coherence and Prolonged Subthalamic Oscillatory Response in the β Band
Source: J Neurosci. 2022 Jun 8;42(23):4681–92. doi: 10.1523/JNEUROSCI.0572-21.2022 (PMC9186803; doi:10.1523/JNEUROSCI.0572-21.2022)
Supplement: Extended Data Table 2-1 — Correlations between β power (different – same), model regressors and behavioral measures. We correlated across participants the changes in β power at each cue (cue “i,” “i + 1”) with behavioral measures (accuracy, reaction time, the number of stimuli sampled, proportion of trials ending on a “same” cue). When correlating trial-wise β power with reaction time or the number of stimuli sampled at the single participant level, we did not find any significant effects. Other than raw power changes, we also included the full GLM regression values from Figure 2 as well as the coherence effects from Figure 5. Note, the listed p-values are uncorrected, and thus the two correlations with p < 0.05 would not survive the correction for multiple comparisons. *If outlier is taken out then correlation is no longer significant (r = 0.47, p = 0.12), see Extended Data Figure 2-1 for reaction time (RT). Outlier detected as more than 1.5 interquartile range above the upper quartile or below the lower quartile, which is appropriate when data is not normally distributed. Download Table 2-1, DOCX file. [file ns-JN-RM-0572-21-s01.docx]

| **Correlations**  (Neural Activity and Behaviour) | **RT** | **# of samples (cues)** | **ACC** | **Proportion end on ‘same’** |
| --- | --- | --- | --- | --- |
| Cue ‘i’ (100-450ms) | r=0.43,p=0.15 | r=0.3,p=0.3 | r=-0.11,p=0.7 | r=-0.15,p=0.6 |
| Cue ‘i+1’ (750-1100ms) | r=-0.49,p=0.09 | r=-0.4,p=0.17 | r=0.45,p=0.12 | r=0.4,p=0.2 |
| Cue ‘i+1’ (1300-1600ms) | r=0.16,p=0.6 | r=0.17,p=0.6 | r=-0.22,p=0.48 | r=-0.1,p=0.7 |
| Regression Model: **Same** 200-350ms  650-800ms | **r=0.62,p=0.025***  r=-0.53,p=0.06 | r=0.53,p=0.06  r=-0.40,p=0.17 | r=-0.14,p=0.6  r=0.22,p=0.5 | r=-0.39,p=0.19  r=0.33,p=0.3 |
| Regression Model: **Evidence** (550-700ms) | r=0.49,p=0.09 | r=0.3,p=0.3 | r=-0.34,p=0.3 | r=-0.39,p=0.2 |
| Regression Model: **Urgency** 0-250ms/500-650ms | r=-0.14,p=0.6 | r=0.06,p=0.8 | r=0.38,p=0.2 | r=0.11,p=0.7 |
| Coherence  510-900ms  900-1240ms | r=-0.34, p=0.25  r=-0.54, p=0.059 | **r=-0.58, p=0.039**  r=-0.20, p=0.5 | r=-0.21, p=0.5  r=0.39, p=0.19 | r=0.29, p=0.34  r=0.17, p=0.6 |
