# Supplementary figures and images for: Conflict Detection in a Sequential Decision Task Is Associated with Increased Cortico-Subthalamic Coherence and Prolonged Subthalamic Oscillatory Response in the β Band
Source: J Neurosci. 2022 Jun 8;42(23):4681–92. doi: 10.1523/JNEUROSCI.0572-21.2022 (PMC9186803; doi:10.1523/JNEUROSCI.0572-21.2022)

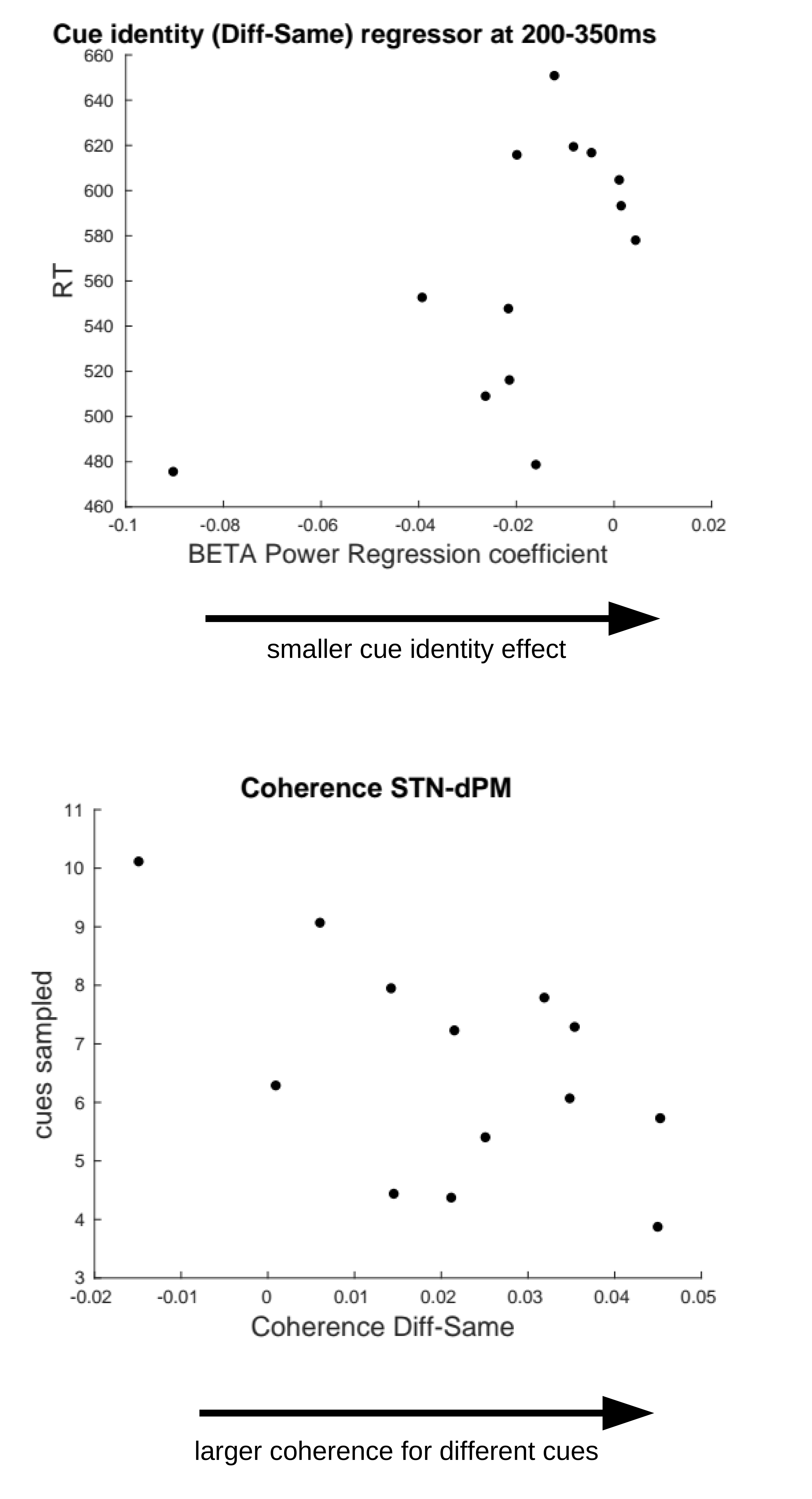

Supplement: Extended Data Figure 2-1 — Correlation between cue identity regressor and reaction time, and between coherence and number of cues sampled Note the p-values associated with these correlations do not survive correction for multiple comparisons. Download Figure 2-1, TIF file. [file ns-JN-RM-0572-21-s02.tif]

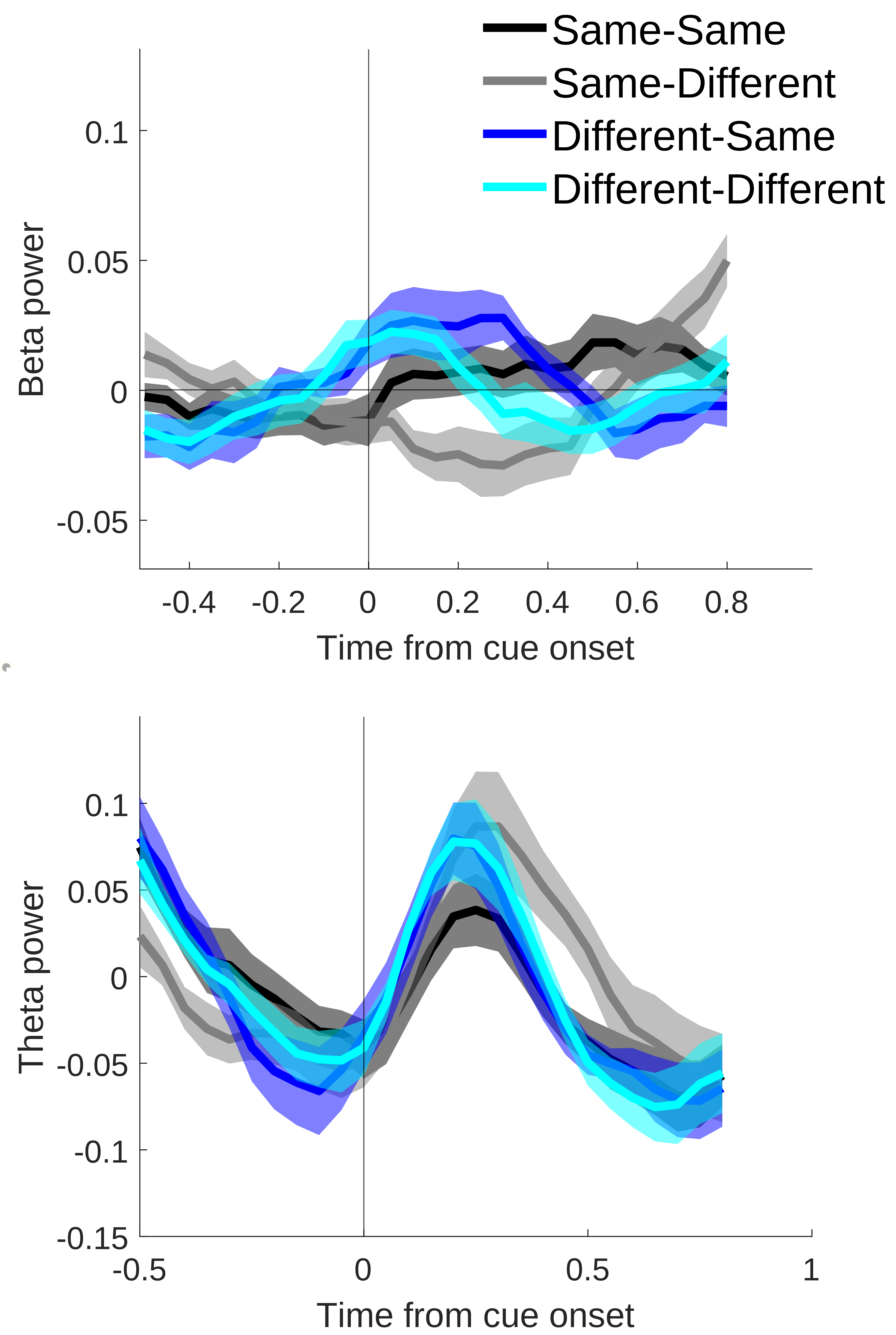

Supplement: Extended Data Figure 3-1 — Effects from Figure 3, plotted with cue i + 1 in detail; for example, “same”-“different” could be a cue sequence: “L-L-R.” Plotted is the response to the last cue of the triplet, “R,” in this example. Top, β Power. Bottom, θ Power. Download Figure 3-1, TIF file. [file ns-JN-RM-0572-21-s03.tif]
